# The complex of *Plasmodium falciparum* falcipain-2 protease with an (E)-chalcone-based inhibitor highlights a novel, small, molecule-binding site

**DOI:** 10.1186/s12936-019-3043-0

**Published:** 2019-12-02

**Authors:** Jonathan M. Machin, Anastassia L. Kantsadi, Ioannis Vakonakis

**Affiliations:** Department of Biochemistry, University of Oxford, South Parks Road, Oxford, OX1 3QU UK

**Keywords:** *Plasmodium*, Falcipain, Inhibitor, Complex, Structure, X-ray crystallography

## Abstract

**Background:**

Malaria kills over 400,000 people each year and nearly half the world’s population live in at-risk areas. Progress against malaria has recently stalled, highlighting the need for developing novel therapeutics. The parasite haemoglobin degradation pathway, active in the blood stage of the disease where malaria symptoms and lethality manifest, is a well-established drug target. A key enzyme in this pathway is the papain-type protease falcipain-2.

**Methods:**

The crystallographic structure of falcipain-2 at 3.45 Å resolution was resolved in complex with an (E)-chalcone small-molecule inhibitor. The falcipain-2–(E)-chalcone complex was analysed with reference to previous falcipain complexes and their similarity to human cathepsin proteases.

**Results:**

The (E)-chalcone inhibitor binds falcipain-2 to the rear of the substrate-binding cleft. This is the first structure of a falcipain protease where the rear of the substrate cleft is bound by a small molecule. In this manner, the (E)-chalcone inhibitor mimics interactions observed in protein-based falcipain inhibitors, which can achieve high interaction specificity.

**Conclusions:**

This work informs the search for novel anti-malaria therapeutics that target falcipain-2 by showing the binding site and interactions of the medically privileged (E)-chalcone molecule. Furthermore, this study highlights the possibility of chemically combining the (E)-chalcone molecule with an existing active-site inhibitor of falcipain, which may yield a potent and selective compound for blocking haemoglobin degradation by the malaria parasite.

## Background

Malaria is estimated to be responsible for 435,000 deaths and over 200 million cases of infection per year, primarily in Africa and Southeast Asia [1]. While huge advancements have been made in reducing both malaria incidence and mortality rates over the last 15 years, more recent data from 2015 to 2017 suggest progress has stalled, highlighting the need for further concentrated research. Particular challenges emerging are resistance to the most effective and commonly used artemisinin-based combination therapy, and deletions of the parasite gene encoding histidine rich protein II, which is a biomarker used in rapid diagnostic tests [1]. There is an ever-growing need for effective new anti-malarials and diagnostic tools to be developed, alongside the goal of a vaccine.

Human-infective parasites of the genus *Plasmodium* invade erythrocytes (e.g., *Plasmodium falciparum*) or reticulocytes (*Plasmodium vivax*) during the malaria blood stage, when symptoms, such as fever, anaemia and inflammation, and lethality occur [2]. While in the intra-erythrocytic stage, *Plasmodium* parasites feed by consuming 60–80% of haemoglobin in red blood cells, breaking it down and using the amino acids as both an energy source and for protein synthesis [3, 4]. An additional purpose for haemoglobin breakdown may be to provide the space needed for the parasite to grow and replicate in the erythrocyte [5]. The breakdown of haemoglobin is carried out in a specialized parasite organelle, the acidic food vacuole, by a ~ 200 kDa protein complex containing cysteine (falcipain-2/2′, FP2/2′), aspartate (plasmepsin II and IV), and histo-aspartic proteases, and a dedicated enzyme (haem detoxification protein) for converting toxic haem into an inert crystalline form (haemozoin) [6]. Genetic analysis has shown many of these components, including FP2, plasmepsin II and the haem detoxification protein, to be essential for parasite viability [7]. Combined, the toxic nature of free haem in solution, the need of the parasite to feed on haemoglobin and to create space for its replication, and the essentiality of many protein components, make the haemoglobin breakdown pathway a strong candidate for anti-malarial therapeutics, as demonstrated by its targeting by current and historic drugs. For example, chloroquine, discovered in 1934 and still used against sensitive malaria strains, binds FP2 and interferes with initial haemoglobin proteolysis [6], as well as haemozoin, where it prevents incorporation of additional haem molecules thereby increasing free haem concentration [8]. Furthermore, in past studies parasite development in cultures was arrested, and malaria treated in murine models, by direct blocking of the haemoglobin breakdown pathway using broad-spectrum cysteine-protease inhibitors [9–11].

Mature FP2 is a 27 kDa papain-type protease responsible for cleaving haemoglobin to small peptides [12], with 93% sequence identity to FP2′ and 68% to parasite falcipain-3. In vitro, FP2/2′ and falcipain-3 are active, at least in part, against the same substrates and can be inhibited by the same small molecule compounds [13]; in addition, genetic studies suggested that in vivo the roles of these proteases may overlap, as FP2 knock-outs could be compensated by increased expression of FP2′ and/or falcipain-3 [9]. As well as having haemoglobinase activity, FP2 is involved in degrading the erythrocyte skeletal protein ankyrin [14] in a process necessary for red blood cell rupture at the end of the parasite intra-erythrocytic cycle. Intriguingly, the optimum pH for FP2-mediated degradation of specific substrates differs, with haemoglobinase activity favoured at pH 5–6 as found in the food vacuole, while ankyrin degradation and FP2 self-activation by autoproteolysis are favoured at neutral or slightly alkaline pH [14]. This suggests that FP2 activity may depend on the local cellular environment, thereby providing a mechanism for substrate discrimination.

A large number of FP2 inhibitors have been identified (e.g., [15–19]) the majority of which are peptide based, although a number of peptidomimetic (e.g., [20]) and non-peptidic inhibitors (e.g., [21, 22]) have also been found. Despite this proliferation of potential therapeutics, no anti-malarials specifically targeting FP2 are currently available. This is in part due to poor selectivity of these inhibitors against human cysteine-proteases of the cathepsin family, which are structurally homologous to FP2. In addition, some of the strongest FP2 inhibitors known are peptides, which limits their potential as drug candidates since they degrade rapidly in vivo and cannot be administered orally. There is substantial interest in understanding the mechanisms of action and binding of non-peptidic FP2 inhibitors, as this knowledge could help in the design of more potent and specific compounds.

In this study, the crystallographic structure of FP2 is determined in complex with an inhibitor from the (E)-chalcone family of molecules [21]. In contrast to previously resolved FP2 or falcipain-3 complexes with small molecules, all of which bound exclusively at the catalytic site [23, 24], this (E)-chalcone inhibitor binds to the rear of the substrate-binding cleft, thereby mimicking interactions seen in FP2 when bound to other proteins [25–27]. As (E)-chalcones can be easily synthesized and derivatised, and possess broadly understood biological properties [28, 29], it is likely that combinations of (E)-chalcone scaffolds with inhibitors targeting the FP2 catalytic site may provide good starting points for anti-malarial drug design.

## Methods

### FP2 cloning, expression and purification

A synthetic DNA fragment (IDT) encoding for mature inactive FP2 (UniProt accession Q9N6S8, residues 245–484, C285A), optimized for expression in *Escherichia coli*, was cloned into plasmid pFloat2 to include an N-terminal His_6_-tag [30]. *Escherichia coli* BL21(DE3) cells transformed with this construct were grown at 37 °C in Luria Broth until OD_600_ of 0.6, upon which isopropyl-β-d-1-thiogalactopyranoside was added to 0.3 mM final concentration to induce protein expression. After overnight incubation at 18 °C cells were harvested and resuspended in 100 mM Tris–Cl (pH 7.4), 10 mM ethylenediaminetetra-acetic acid (EDTA) buffer.

Initial FP2 purification via inclusion body preparation was performed as described previously [9]. Briefly, cell suspensions were lysed by sonication and lysates were centrifuged at 50,000*g* and 4 °C. Lysate pellets were washed twice in 20 mM Tris–Cl (pH 8), 2.5 M urea, 2.5% v/v Triton X-100 buffer and twice in 20 mM Tris–Cl (pH 8), 20% w/v sucrose buffer. The resultant pellets were solubilized in 6 M guanidine-HCl, 20 mM Tris–Cl (pH 8), 500 mM NaCl, 10 mM imidazole buffer at room temperature. The suspension was loaded onto a HisTrap Ni^2+^-affinity column (GE) equilibrated in solubilization buffer. The column was washed with 8 M urea, 20 mM Tris–Cl (pH 8), 500 mM NaCl, 30 mM imidazole buffer prior to protein elution in the same buffer with 1 M imidazole. FP2-containing fractions were pooled and concentrated to 2 mg/ml using spin ultrafiltration. Protein concentration was calculated by UV absorption at 280 nm and an extinction coefficient estimated from the amino acid sequence [31].

FP2 samples were reduced by the addition of dithiothreitol at 10 mM final concentration and incubation at 37 °C for 45 min. FP2 was then refolded by 100× rapid dilution into 100 mM Tris–Cl (pH 9), 1 mM EDTA, 30% v/v glycerol, 0.25 M arginine, 1 mM reduced glutathione and 1 mM oxidized glutathione buffer while stirring, and incubated at 4 °C for 20 h. The refolded protein was then filtered (0.22 μm polyethersulphone membrane, Millipore) and concentrated to 1 mg/ml, during which a 10× dilution with deionized water was carried out. Protein samples were applied to an anion exchange column (HiTrap-Q, GE) equilibrated with 20 mM Tris–Cl (pH 7.5) buffer and eluted with a linear gradient of 1 M NaCl. FP2-containing fractions were pooled and concentrated to 2 mg/ml, during which the buffer was exchanged to 2.5 mM Tris–Cl (pH 7.5), 5 mM NaCl. Final purification was carried out by size exclusion chromatography using a HiLoad 75/600 Superdex 200 column (GE) equilibrated with 2.5 mM Tris–Cl (pH 7.5), 5 mM NaCl. FP2-containing fractions were pooled and concentrated by spin ultrafiltration.

### FP2 crystallization, data collection and refinement

FP2 was crystallized using protein samples at 7 mg/ml and the sitting drop method. Two-hundred nanoliter-size drops with 1:1 and 1.3:0.7 protein-to-mother liquor ratios were set up and incubated at 18 °C. FP2 crystals were obtained using a mother liquor containing 0.12 M of monosaccharide mixture (d-glucose; d-mannose; d-galactose; l-fucose; d-xylose; *N*-acetyl-d-glucosamine), 0.1 M Tris/Bicine (pH 8.5); 20% v/v glycerol, 10% w/v polyethylene glycol 4000. Crystals appeared after a week of incubation and continued to evolve over a 6-week period. (E)-chalcone inhibitors [21] #48 ((E)-3-(benzo[d] [1, 3] dioxol-5-yl)-1-(3-nitrophenyl)prop-2-en-1-one, EC48) and #54 (1-(3-nitrophenyl)-3-(2,4,5-trimethoxyphenyl)prop-2-en-1-one, EC54), sourced from ChemBridge, were dissolved in 100% dimethylsulphoxide to a concentration of 100 mM and diluted to 1 mM in the crystallization mother liquor. FP2 crystals were then soaked in EC48- or EC54-containing mother liquor for 80–190 min, harvested in nylon loops and flash-cooled in liquid nitrogen.

X-ray diffraction data were collected at beamline I03 of the Diamond Light Source (Harwell, UK) at 100 K; however, only EC48-soaked FP2 crystals produced usable data to 3.45 Å maximum resolution. Crystallographic data collection and refinement statistics are shown in Table 1. The space group was determined to be P 3_2_ 2 1 with two copies of FP2 per asymmetric unit of the crystals. Data were processed with autoPROC [32] and STARANISO [33] and solved by molecular replacement using PHASER [34] and a previous, 2.2 Å-resolution crystallographic structure of FP2 (PDB ID 2OUL, [26]) as molecular replacement model. The structure was iteratively refined using Buster version 2.10.3 [35] and Coot [36]. The previous crystallographic structure of FP2 was used as external target restraint throughout Buster refinement. Geometry restraints for EC48 were generated using Grade [37]. OMIT-type maps were generated in Buster by refining the FP2 structure in the absence of EC48 and excluding the volume occupied by the inhibitor from bulk solvent flattening. Molprobity was used to assess model quality [38]. PyMOL was used for graphical representations [39]. The FP2 C285A mutation was reverted in PyMOL for illustration purposes. The crystallographic structure and underpinning data of the FP2–EC48 complex have been deposited in the RCSB Protein Data Bank under Accession number 6SSZ.

**Table 1:** Crystallographic data and refinement statistics

Protein name	Falcipain-2–EC48 complex
RCSB ID	6SSZ
*Data collection statistics*	
Beamline	DLS/I03
Wavelength (Å)	0.9763
Space group	P 3_2_ 2 1
Unit cell (Å,°)	109.43 109.43 107.25, 90 90 120
Resolution range (Å)^a^	94.77–3.45 (3.85–3.45)
Diffraction limits (principal axes, Å)^a^	4.375 4.375 3.292
R_merge_ (I)^a^	0.204 (2.310)
R_meas_ (I)^a^	0.209 (2.378)
R_pim_ (I)^a^	0.048 (0.554)
Completeness (spherical, %)^a^	59.3 (10.9)
Completeness (ellipsoidal, %)^a^	90.2 (66.9)
Multiplicity^a^	18.9 (17.6)
I/s^a^	10.7 (1.5)
CC_½_^a^	1.00 (0.44)
*Refinement statistics*	
R_work_ (reflections)	0.255 (5712)
R_free_ (reflections)	0.308 (288)
*Number of atoms*	
Protein	3793
Ligands	44
*Average B factors (Å*^*b*^*)*	
Protein	147.5
Ligands	162.4
*RMSD from ideal*	
Bonds/angles (Å/º)	0.009/1.14
*MolProbity statistics*^b^	
Ramachandran favoured (%)	97.5%
Ramachandran disallowed (%)	0
Rotamers favoured (%)	98.8%
Poor rotamers (%)	0
Clashscore^c^	5.48 (100th percentile)
MolProbity score^c^	1.40 (100th percentile)

^a^By STARANISO [33]. Values in parenthesis correspond to the highest resolution shell

^b^Calculated by the MolProbity online server [38]

^c^100th percentile is the best among structures of comparable resolution; 0th percentile is the worst

### Sequence analysis

Amino acid sequences were obtained from the UniProt database and correspond to the following Accession numbers: human cathepsin B, P07858; human cathepsin K, P43235; human cathepsin L1, P07711; *P. falciparum* falcipain-1, Q8I6V0; *P. falciparum FP2*, Q9N6S8; *P. falciparum FP2′*, Q8I6U5; *P. falciparum* falcipain-3, Q8IIL0. Sequences were aligned by Clustal Omega [40].

## Results

### Crystallographic structure of an (E)-chalcone inhibitor bound to FP2

**Fig. 1:**
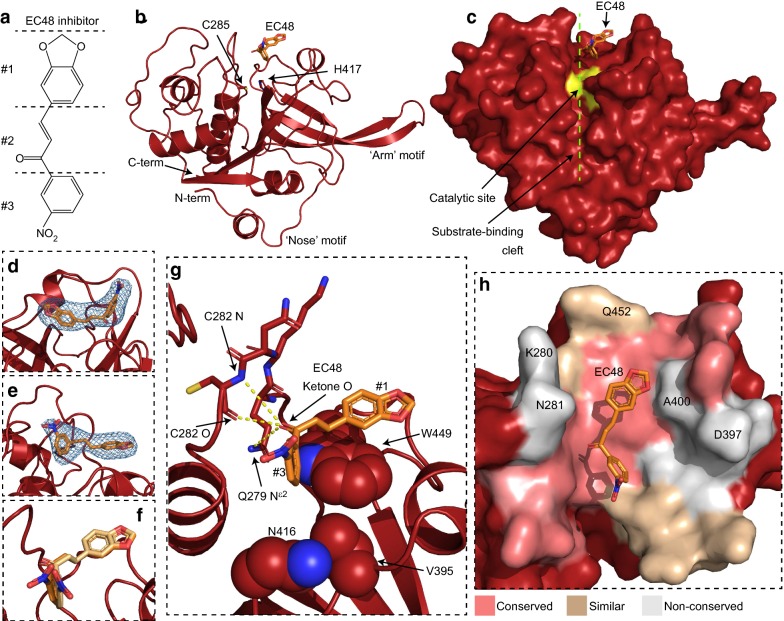
Structural mode of EC48 binding to FP2. **a** Molecular structure of the EC48 inhibitor. The different chemical groups of EC48 are demarcated as #1, benzodioxol; #2, ketone; #3, nitrobenzene. **b** A single FP2 copy from the crystallographic structure, shown in schematic representation (red). EC48 (orange) and the side chains of the catalytic dyad residues (C285, H417) are shown as sticks. **c** Surface representation of FP2 in the same orientation as panel **b**. The surface of catalytic site residues is coloured yellow. The substrate-binding cleft is denoted via a dashed line (green). **d**, **e** Sections of OMIT-type F_o_–F_c_ electron density maps showing the EC48 binding sites of the two FP2 copies in the crystal, contoured at 3.5 σ. EC48 ligands were absent during OMIT refinement and are shown here for reference. **f** Superposition of EC48 binding positions in the two FP2 copies in the crystal. **g** Molecular interactions involved in EC48 binding. The side chains of FP2 residues that form Van der Waals and p-stacking interactions with EC48 are shown as spheres. FP2 residues involved in hydrogen bonding to EC48 are shown as sticks and putative hydrogen bonds are denoted by dashed lines in yellow. **h** Sequence conservation at the vicinity (< 10 Å distance) of the EC48 binding site, shown in surface representation. FP2 residues conserved in > 50% of human cathepsin sequences in the alignment of Fig. 2 are coloured pink. Residues conserved or similar in > 50% of human cathepsins are coloured light brown. FP2 residues that differ in > 50% of human cathepsins are coloured grey. FP2 residues noted in text for their divergence in cathepsins are labelled

Crystals of recombinant mature FP2, lacking the inhibitory pro-domain and with the catalytic site cysteine substituted by alanine for increased stability [12], were soaked with two previously identified (E)-chalcone molecules [21], EC54, which exhibited the most potent purely competitive inhibition among derivatives in this earlier study, and EC48. Enzymatic assays showed EC48 as affecting both FP2 substrate binding and enzyme activity (mixed inhibition), with IC_50_ value of 8.5 ± 0.8 mM [21]. Crystals derivatized with EC48 (Fig. 1a), diffracted anisotropically to 3.45 Å maximum resolution (Table 1); FP2 crystals soaked with EC54 did not yield usable crystallographic data. The FP2-EC48 structure, resolved by molecular replacement, revealed two FP2 copies per asymmetric unit of the crystal. Both protein copies exhibited bound EC48 inhibitor (see below) and had nearly identical tertiary structure, with root-mean-square-deviation (RMSD) of C_a_ atoms below 0.1 Å. Furthermore, the FP2 structure under these conditions was highly similar to that obtained by previous crystallographic analyses of this protease, with C_a_ RMSDs varying between 0.21 Å (compared to FP2 in complex with the protein-based inhibitor chagasin [26]) and 0.43 Å (compared to FP2 in complex with the small molecule inhibitor E64 [23]). This suggests that EC48 binding does not induce large-scale structural rearrangement of FP2.

Similar to other members of the papain protease family, FP2 comprises two distinct sub-domains with primarily α-helical and β-stranded composition, respectively (Fig. 1b). The substrate cleft and the catalytic site are located between these sub-domains. Compared to papain-type proteases mature FP2 features a 14-residue long N-terminal extension and an insertion at the β6-β7 loop (Fig. 1b). The latter, known as the ‘arm’ motif, participates in haemoglobin binding, whereas the N-terminal extension, the ‘nose’ motif, is suggested to enable proper folding of FP2. These features are conserved among papain-type proteases of malaria parasites, but not found in any others, including the human homologues cathepsin K, L and B [11, 12, 23–27].

Immediately following molecular replacement with the apo-FP2 structure, strong residual electron density was observed in the FP2 substrate cleft, consistent with small molecule binding. This residual electron density became clearer upon FP2 refinement (Fig. 1d, e) and allowed for the unambiguous placement of EC48 (Fig. 1b, c), which adopts highly similar placements and conformations in both FP2 copies of the crystal (Fig. 1f). Although chalcones are often depicted as planar molecules (Fig. 1a), the relative orientation of the two aromatic groups can vary; indeed, in previously resolved crystallographic structures of protein–chalcone complexes the relative angle between the two rings spanned values between 21° (PDB ID 5YX4) and 88° (PDB ID 5EZP). In the FP2-EC48 complex the (E)-chalcone adopted a nearly perpendicular arrangement between the two rings, with relative angles of 82° and 89° in the two copies of the inhibitor in the crystal.

### Analysis of the (E)-chalcone-FP2 binding mode

**Fig. 2:**
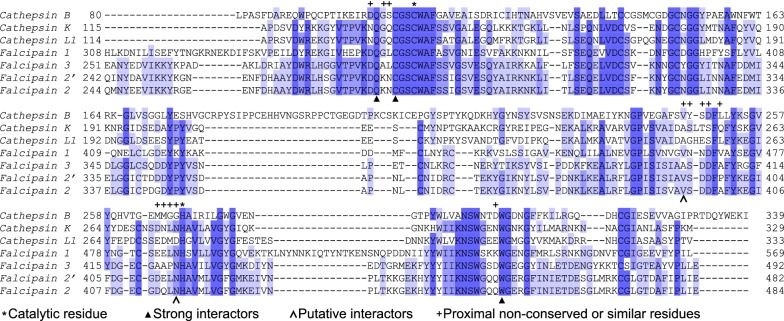
Alignment of *Plasmodium falciparum* falcipain and human cathepsin proteases. Sequence alignment of mature parasite falcipain and human cathepsin proteases. Sequence numbering derives from UniProt entries as described in “Methods”. Darker blue shading denotes increased conservation for a given amino acid position. Above alignments asterisks mark residues of the catalytic dyad, and crosses mark non-conserved and similar residues in the vicinity of the EC48 binding site (coloured light brown and grey in Fig. 1h). Below alignments arrowheads denote residues that participate in strong (filled arrowheads) or putative (open arrowheads) interactions with EC48

EC48 locates in a position distinct from the FP2 catalytic site (Fig. 1c). Analysis of the structure suggests that EC48 binding is mediated by hydrophobic and π-stacking interactions between the benzodioxol group of the inhibitor (demarcated #1 in the inhibitor structure, Fig. 1a) and the W449 sidechain of FP2 (Fig. 1g). Further, the inhibitor ketone O (#2 in Fig. 2a) is oriented in a manner that would allow hydrogen bonding to the FP2 C282 backbone O and N, and the Q279 sidechain Nε^2^, although the distances between these atoms (3.7, 4.3 and 4.4 Å, respectively) suggest any hydrogen bonds would be weak and mostly electrostatic in character [41]. The inhibitor nitrobenzene group (#3 in Fig. 1a) was slightly mobile in the crystals (Fig. 1f), with its benzene moiety participating in limited Van der Walls contacts with the sidechains of FP2 V395 and N416 in one of the two copies of the complex; the inhibitor nitro moiety did not contact the protein.

The binding modality observed in the FP2–EC48 complex is in agreement with previous spectroscopic analysis that showed reduction of intrinsic tryptophan fluorescence upon EC48 binding [21], likely due to EC48 interaction with FP2 W449 (Fig. 1g). In addition, the structure provides a mechanism for the observed mixed inhibition of FP2 by EC48, as the molecule occupies part of the substrate binding cleft, thereby competing with substrates, and locates just 5.1 and 7.5 Å away from the FP2 H417 and C285, respectively, which comprise the catalytic dyad (Fig. 1b). Thus, it is highly likely that EC48 binding alters the conformational dynamics of the catalytic site, thereby affecting the kinetics of proteolysis.

FP2 residues that form the strongest interactions with EC48 in the crystal are strictly conserved in FP2′ and falcipain-3 but also in human cathepsins (Fig. 2), suggesting that this inhibitor likely would not discriminate between parasite and host proteases. Nevertheless, structural and sequence analyses suggest that several solvent-exposed amino acids proximal to, but not interacting with, EC48 are not conserved between falcipains and cathepsins (Figs. 1h and 2). Such falcipain residues could be targeted, and the inhibitor interaction made more specific for these malaria parasite proteases, by modifying EC48 to interact with the respective side chains. Notably, FP2 amino acids K280, N281, D397, A400, and Q452 (Figs. 1h and 2), situated between 4 and 10 Å away from EC48, have equivalents with substantially different chemical properties (e.g., K280 → G; N281 → S; D397 → S/T, A400 → L/Q, Q452 → E/D) in many human cathepsins. Thus, EC48 inhibitor may serve as basis for modifications that would yield highly selective small molecules.

## Discussion

### Comparison of EC48 binding to previously resolved falcipain inhibitors

**Fig. 3:**
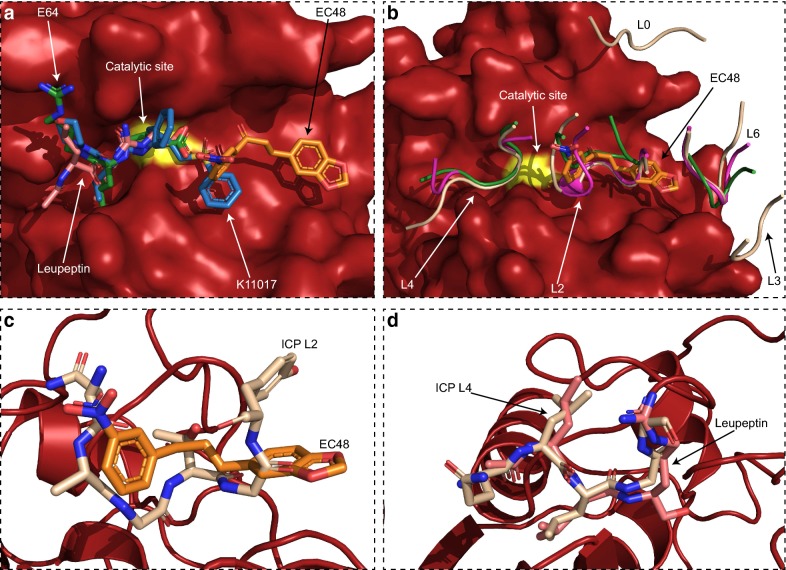
Comparison of EC48 binding to other FP2 inhibitors. **a** Superposition of small molecule inhibitors resolved in complex with *Plasmodium* parasite falcipain proteases on the FP2 structure of this study (surface representation, red; catalytic site C285 coloured yellow). Inhibitors are shown as sticks. EC48 (orange) from the present study; K11017 (blue) from PDB 3BWK; leupeptin (pink) from PDB 3BPM; E64 (green) from PDB 3BPF. **b** Superposition of protein-based inhibitors of falcipain (schematic representation) on the FP2 structure of this study (surface, as in panel **a**). Only the falcipain-interacting loops of these inhibitors are shown for clarity. Loops are numbered according to ICP nomenclature [27] and coloured light brown (ICP; PDB 3PNR), magenta (chagasin; PDB 2OUL) or green (cystatin; PDB 1YVB). EC48 (orange, sticks) from the present study is shown as reference. **c** Overlay of the EC48 (orange, sticks) binding position on FP2 with that of L2 from ICP (light brown, sticks). **d** Similar overlay of the leupeptin (pink) and ICP L4 (light brown) binding positions

The malaria FP2 protease is a key target for the development of small molecule inhibitors, which may form the basis of anti-malarials. This study presented the FP2 structure in complex with a mixed-type inhibitor, EC48. FP2 was previously resolved in complex with an irreversible small molecule inhibitor of papain-type proteases, E64 [23], while the falcipain-3 structure was determined in complex with the small molecule inhibitor K11017 [23] and the peptidomimetic leupeptin [24]. In all cases, the inhibitory molecule interacts with the protease at the catalytic site and immediately in front of it along the substrate cleft (Fig. 3a). Only K11017 was seen to form contacts with the middle of the cleft. In contrast, EC48 occupies the middle of the substrate cleft via its nitrobenzene group and binds to the rear of the cleft via its benzodioxol group. Thus, EC48 interacts with a distinct FP2 surface that was not previously bound by inhibitors in structures of falcipain complexes. Further, the position of EC48 away from the catalytic site raises the possibility that it could be combined with a catalytic site-binding molecule, leading to increased inhibitor potency and specificity. For example, the nitrobenzene group of EC64 and the sulfonylbenzene group of K11017 are very close in space, with average interatomic distance of 3 Å albeit with different orientations (Fig. 3a); thus, linking the two molecules via the benzene moiety provides a clear avenue for future organic synthesis efforts.

In vivo, undesired action of papain-type proteases is blocked by forming complexes with other proteins, such as the inhibitor of cysteine proteases (ICP) family found in *Plasmodium* [42, 43]. ICPs are known to interfere with host protease activity to block host cell apoptosis, but are also highly potent against FP2 and other parasite cysteine proteases to avoid indiscriminate proteolytic damage during erythrocyte rupture [43]. Three complexes of *P. falciparum* FP2 with cystatin [25], a classical cysteine protease inhibitor, chagasin [26], an inhibitor from *Trypanosoma cruzi*, and the ICP of *Plasmodium berghei* [27], have previously been resolved. Although cystatin is structurally distinct from chagasin and ICP, their FP2 interactions are surprisingly similar suggesting that cysteine protease inhibitors have emerged through convergent evolution. Specifically, protein inhibitors interact with the FP2 substrate cleft via a series of b-strand-connecting loops (Fig. 3b), numbered (from front to back of the cleft) as L4, L2 and L6 [25–27]. ICPs form additional FP2 interactions, compared to cystatin and chagasin, away from the substrate cleft via L0 and L3, which may account for their higher affinity and specificity for *Plasmodium* parasite proteases [27]. Comparing the EC48 inhibitor to these protein complexes shows that its FP2 binding site primarily corresponds to that of L2 and also partly spans the gap towards L6. Notably, despite EC48 having no chemical similarity to peptides, its arrangement in space follows closely that of the L2 peptide backbone (Fig. 3c). This type of close correspondence between the binding positions of small molecules and natural complex partners is often seen in potent inhibitors, such as in leupeptin that follows closely the orientation of L4 from inhibitory proteins (Fig. 3d) and has sub-nanomolar K_i_ for papain-type proteases [23]. Thus, it is likely that elaborating on the EC48 molecular structure, while seeking to mimic the natural protein inhibitors, has the potential to improve its interactions with FP2 and increase its binding strength and specificity.

## Conclusions

The crystallographic structure of FP2 with a previously unresolved inhibitor, EC48, was determined to a resolution of 3.45 Å. The binding mode of EC48 provides a mechanistic explanation for the mixed inhibition of FP2 by this molecule seen in previous biochemical assays. The structure shows that EC48 binds only to the rear of the FP2 substrate cleft, thereby mimicking interactions formed by the L2 loop of naturally occurring, protein-based inhibitors of FP2. This binding site is novel among small molecule falcipain inhibitors resolved to date and offers clear avenues for chemical synthesis to elaborate EC48 towards a more potent and selective molecule. Particularly noteworthy are the sequence divergence between falcipains and human cathepsin proteases around this binding site, and the possibility of combining EC48 with an existing falcipain catalytic site inhibitor. Knowledge about the binding site and interactions of EC48 will facilitate the search for anti-malarials targeting FP2, especially as it is based around the medically privileged chemical framework of (E)-chalcones.

## Data Availability

The structural model and underlying crystallographic structure factors used for model calculation are available at the RCSB Protein Data Bank under Accession number 6SSZ.

## Abbreviations

FP2: falcipain-2
FP2′: falcipain-2′
EDTA: ethylenediaminetetraacetic acid
EC48: (E)-3-(benzo[d][1, 3]dioxol-5-yl)-1-(3-nitrophenyl)prop-2-en-1-one
EC54: 1-(3-nitrophenyl)-3-(2,4,5-trimethoxyphenyl)prop-2-en-1-one
RMSD: root mean square deviation
ICP: inhibitor of cysteine proteases

## References

[1] WHO (2018). World malaria report 2018.

[2] Kirchgatter K, Del Portillo HA (2005). Clinical and molecular aspects of severe malaria. An Acad Bras Cienc.

[3] Sherman IW (1977). Amino acid metabolism and protein synthesis in malarial parasites. Bull World Health Organ.

[4] Sherman IW, Tanigoshi L (1970). Incorporation of ^14^C-amino acids by malaria (*Plasmodium lophurae*). IV. In vivo utilization of host cell haemoglobin. Int J Biochem.

[5] Francis SE, Sullivan DJ, Goldberg DE (1997). Hemoglobin metabolism in the malaria parasite *Plasmodium falciparum*. Annu Rev Microbiol.

[6] Chugh M, Sundararaman V, Kumar S, Reddy VS, Siddiqui WA, Stuart KD, Malhotra P (2013). Protein complex directs hemoglobin-to-hemozoin formation in *Plasmodium falciparum*. Proc Natl Acad Sci USA.

[7] Zhang M, Wang C, Otto TD, Oberstaller J, Liao X, Adapa SR (2018). Uncovering the essential genes of the human malaria parasite *Plasmodium falciparum* by saturation mutagenesis. Science.

[8] Hempelmann E (2007). Hemozoin biocrystallization in *Plasmodium falciparum* and the antimalarial activity of crystallization inhibitors. Parasitol Res.

[9] Rosenthal PJ, Lee GK, Smith RE (1993). Inhibition of a *Plasmodium vinckei* cysteine proteinase cures murine malaria. J Clin Invest.

[10] Olson JE, Lee GK, Semenov A, Rosenthal PJ (1999). Antimalarial effects in mice of orally administered peptidyl cysteine protease inhibitors. Bioorg Med Chem.

[11] Teixeira C, Gomes JR, Gomes P (2011). Falcipains, *Plasmodium falciparum* cysteine proteases as key drug targets against malaria. Curr Med Chem.

[12] Hogg T, Nagarajan K, Herzberg S, Chen L, Shen X, Jiang H (2006). Structural and functional characterization of Falcipain-2, a hemoglobinase from the malarial parasite *Plasmodium falciparum*. J Biol Chem.

[13] Ramjee MK, Flinn NS, Pemberton TP, Quibell M, Wang Y, Watts JP (2006). Substrate mapping and inhibitor profiling of falcipain-2, falcipain-3 and berghepain-2: implications for peptidase anti-malarial drug discovery. Biochem J.

[14] Hanspal M, Dua M, Takakuwa Y, Chishti AH, Mizuno A (2002). *Plasmodium falciparum* cysteine protease falcipain-2 cleaves erythrocyte membrane skeletal proteins at late stages of parasite development. Blood.

[15] Royo S, Schirmeister T, Kaiser M, Jung S, Rodriguez S, Bautista JM, Gonzalez FV (2018). Antiprotozoal and cysteine proteases inhibitory activity of dipeptidyl enoates. Bioorg Med Chem.

[16] Chen W, Huang Z, Wang W, Mao F, Guan L, Tang Y (2017). Discovery of new antimalarial agents: second-generation dual inhibitors against FP-2 and PfDHFR via fragments assembely. Bioorg Med Chem.

[17] Melo PMS, El Chamy Maluf S, Azevedo MF, Paschoalin T, Budu A, Bagnaresi P (2018). Inhibition of *Plasmodium falciparum* cysteine proteases by the sugarcane cystatin CaneCPI-4. Parasitol Int.

[18] Ang KK, Ratnam J, Gut J, Legac J, Hansell E, Mackey ZB (2011). Mining a cathepsin inhibitor library for new antiparasitic drug leads. PLoS Negl Trop Dis.

[19] Salas-Sarduy E, Guerra Y, Covaleda Cortes G, Aviles FX, Chavez Planes MA (2017). Identification of tight-binding plasmepsin II and falcipain 2 inhibitors in aqueous extracts of marine invertebrates by the combination of enzymatic and interaction-based assays. Mar Drugs.

[20] Nizi E, Sferrazza A, Fabbrini D, Nardi V, Andreini M, Graziani R (2018). Peptidomimetic nitrile inhibitors of malarial protease falcipain-2 with high selectivity against human cathepsins. Bioorg Med Chem Lett.

[21] Bertoldo JB, Chiaradia-Delatorre LD, Mascarello A, Leal PC, Cordeiro MN, Nunes RJ (2015). Synthetic compounds from an in house library as inhibitors of falcipain-2 from *Plasmodium falciparum*. J Enzyme Inhib Med Chem.

[22] Hernandez-Gonzalez JE, Salas-Sarduy E, Hernandez Ramirez LF, Pascual MJ, Alvarez DE, Pabon A (2018). Identification of (4-(9H-fluoren-9-yl) piperazin-1-yl) methanone derivatives as falcipain 2 inhibitors active against *Plasmodium falciparum* cultures. Biochim Biophys Acta Gen Subj.

[23] Kerr ID, Lee JH, Pandey KC, Harrison A, Sajid M, Rosenthal PJ, Brinen LS (2009). Structures of falcipain-2 and falcipain-3 bound to small molecule inhibitors: implications for substrate specificity. J Med Chem.

[24] Kerr ID, Lee JH, Farady CJ, Marion R, Rickert M, Sajid M (2009). Vinyl sulfones as antiparasitic agents and a structural basis for drug design. J Biol Chem.

[25] Wang SX, Pandey KC, Somoza JR, Sijwali PS, Kortemme T, Brinen LS (2006). Structural basis for unique mechanisms of folding and hemoglobin binding by a malarial protease. Proc Natl Acad Sci USA.

[26] Wang SX, Pandey KC, Scharfstein J, Whisstock J, Huang RK, Jacobelli J (2007). The structure of chagasin in complex with a cysteine protease clarifies the binding mode and evolution of an inhibitor family. Structure.

[27] Hansen G, Heitmann A, Witt T, Li H, Jiang H, Shen X (2011). Structural basis for the regulation of cysteine-protease activity by a new class of protease inhibitors in *Plasmodium*. Structure.

[28] Zhuang C, Zhang W, Sheng C, Zhang W, Xing C, Miao Z (2017). Chalcone: a privileged structure in medicinal chemistry. Chem Rev.

[29] Gomes MN, Muratov EN, Pereira M, Peixoto JC, Rosseto LP, Cravo PVL (2017). Chalcone derivatives: promising starting points for drug design. Molecules.

[30] Rogala KB, Dynes NJ, Hatzopoulos GN, Yan J, Pong SK, Robinson CV (2015). The *Caenorhabditis elegans* protein SAS-5 forms large oligomeric assemblies critical for centriole formation. Elife.

[31] Pace CN, Vajdos F, Fee L, Grimsley G, Gray T (1995). How to measure and predict the molar absorption coefficient of a protein. Protein Sci.

[32] Vonrhein C, Flensburg C, Keller P, Sharff A, Smart O, Paciorek W (2011). Data processing and analysis with the autoPROC toolbox. Acta Crystallogr D Biol Crystallogr.

[33] Tickle IJ, Flensburg C, Keller P, Paciorek W, Sharff A, Vonrhein C, Bricogne G (2018). STARANISO.

[34] McCoy AJ, Grosse-Kunstleve RW, Adams PD, Winn MD, Storoni LC, Read RJ (2007). Phaser crystallographic software. J Appl Crystallogr.

[35] Bricogne G, Blanc E, Brandl M, Flensburg C, Keller P, Paciorek W (2017). BUSTER.

[36] Emsley P, Lohkamp B, Scott WG, Cowtan K (2010). Features and development of Coot. Acta Crystallogr D Biol Crystallogr.

[37] Smart OS, Womack TO, Sharff A, Flensburg C, Keller P, Paciorek W (2011). GRADE.

[38] Chen VB, Arendall WB, Headd JJ, Keedy DA, Immormino RM, Kapral GJ (2010). MolProbity: all-atom structure validation for macromolecular crystallography. Acta Crystallogr D Biol Crystallogr.

[39] DeLano WL (2002). The PyMOL Molecular Graphics System.

[40] Sievers F, Wilm A, Dineen D, Gibson TJ, Karplus K, Li W (2011). Fast, scalable generation of high-quality protein multiple sequence alignments using Clustal Omega. Mol Syst Biol.

[41] Jeffrey GA (1997). An introduction to hydrogen bonding.

[42] Rennenberg A, Lehmann C, Heitmann A, Witt T, Hansen G, Nagarajan K (2010). Exoerythrocytic *Plasmodium* parasites secrete a cysteine protease inhibitor involved in sporozoite invasion and capable of blocking cell death of host hepatocytes. PLoS Pathog.

[43] Pandey KC, Singh N, Arastu-Kapur S, Bogyo M, Rosenthal PJ (2006). Falstatin, a cysteine protease inhibitor of *Plasmodium falciparum*, facilitates erythrocyte invasion. PLoS Pathog.

